# *In vitro* assays reveal inherently insecticide-tolerant termite symbionts

**DOI:** 10.3389/fphys.2023.1134936

**Published:** 2023-07-12

**Authors:** Alison G. Blanton, Samontriona Perkins, Brittany F. Peterson

**Affiliations:** Department of Biological Sciences, Southern Illinois University Edwardsville, Edwardsville, IL, United States

**Keywords:** symbiont, insect, insecticide resistance, microbiota, imidacloprid

## Abstract

**Introduction:** Termite symbionts are well known for conferring a myriad of benefits to their hosts. Bacterial symbionts are repeatedly associated with increased fitness, nutritional supplementation, pathogen protection, and proper development across insect taxa. In addition, several recent studies link bacterial symbionts to reduced insecticide efficacy. This has important implications both in pest control management and environmental bioremediation efforts. Insects’ guts may be a valuable resource for microbes with broad application given their unique niches and metabolic diversity. Though insecticide resistance in termites is considered unlikely due to their life history, the close association of termites with a multitude of bacteria raises the question: is there potential for symbiont-mediated pesticide tolerance in termites?

**Methods and results:** We identified a candidate that could grow in minimal medium containing formulated pesticide. This bacterial isolate was then subjected to continuous culture and subsequently demonstrated improved performance in the presence of pesticide. Isolates subjected to continuous culture were then grown at a range of concentrations from 1–10X the formulation rate. After constant exposure for several generations, isolates grew significantly better.

**Conclusion:** Here we demonstrate that naïve insect hosts can harbor symbionts with inherent insecticide tolerance capable of rapid adaptation to increasing insecticide concentrations overtime. This has broad implications for both pest control and environmental cleanup of residual pesticides.

## 1 Introduction

### 1.1 Usefulness of pesticides and the growing threat of resistance

Pesticides and herbicides are some of the most common agricultural tools in use (Kalia and Gosal, 2011). A broad range of chemistries are used to control pests and prevent crop loss (Kalia and Gosal, 2011; Fernandez-Cornejo et al., 2014). These chemical applications are largely successful with many crop yields in the United States holding steady or increasing over the last several decades (Fernandez-Cornejo et al., 2014). However, continued reliance on these tools comes with safety risks including negative impacts on off-target organisms, accumulation in the environment, and reduced efficacy due to target resistance (Burke et al., 2018; Gregorc et al., 2018; Eng et al., 2019). Though pesticide rotation is a standard recommendation, it is not always feasible due to factors such as cost, safety risks, accessibility, or target susceptibility (Dara, 2019). Additionally, some chemical pesticides have long half-lives resulting in environmental accumulation and prolonged target exposure (Wagner, 2016).

Pesticide technology evolves to align with needs related to management practices, environmental consciousness, and pest resistance, but the root the mission of bettering the health of a society or increasing the crop yields has stayed constant (Zadoks and Waibel, 2000; Leoci and Ruberti, 2021). Poor stewardship of pesticides has contributed to the development of pesticide resistance within a population, but selection on the insect host is just one mechanism of pesticide resistance (Berticat et al., 2002.; Kikuchi et al., 2012.; Pietri et al., 2018). Recently symbiont-mediated detoxification of pesticides has emerged as an important consideration in the arms race against pests (Kikuchi et al., 2012). Some insect gut symbionts can break down insecticides within their host thereby increasing host survival rates (Kikuchi et al., 2012; Pang et al., 2018). This phenomenon has been documented in several insect taxa associated with a variety of bacteria partners (Kikuchi et al., 2012; Soltani et al., 2017; Pietri et al., 2018; Xia et al., 2018). While this association is detrimental in efforts to control pests, it also has important implications for bioremediation. Given the natural association bacteria with insect hosts and the ability of some bacteria to metabolize insecticides, insect symbionts should be prime candidates for solutions to pesticide resistance and a faster approach to bioremediation.

### 1.2 Bioremediation

Bioremediation is the use of microorganisms to consume or breakdown environmental pollutants to aid in the rehabilitation of polluted areas (Sharma et al., 2018; Dutta et al., 2021). There are two main types of bioremediation techniques, *in situ* and *ex situ*. (Parween et al., 2018). *Ex situ* bioremediation is when soil or water is brought to a worksite and inoculated to treat and decontaminate the environmental substrate (Tomei and Daugulis, 2013). *In situ* bioremediation is when microorganisms are engineered and brought to the site from an external source and added to the contaminated area (Pandey et al., 2009). In both *in situ* and *ex situ* bioremediation efforts, contaminated waste is biotransformed by microorganisms if their growth conditions are met (Vidali et al., 2011). Matching the specific growth requirements for each microbe with the conditions in the contaminated environment can make for a tedious task. One method of streamlining this process in bioaugmentation. Bioaugmentation is the use bioengineering to increase the efficacy with which microbes are able to process a contaminant (Pandey et al., 2009; Parween et al., 2018; Dutta et al., 2021). Bioaugmented organisms have been used in recent years to help clean up oil spills and reduce environmental abundance of microplastics (Arora, 2018).

The future pesticide bioremediation relies on a better understanding of microbes with inherent tolerance or metabolic ability including the abiotic factors that contribute to their efficacy (Gentry et al., 2004). Because these microbes are typically identified due to their association with polluted environments, such as agricultural fields or sites of runoff, little is known about the ideal growth conditions or preferred carbon/nitrogen sources (Singh et al., 2004). Importantly, bacteria often thrive in concert with other microbes making cultivation of a single isolate complex. One potential source of these microbes that has only recently been identified is in association with targeted pest insects (Kikuchi et al., 2012; Soltani et al.,2017; Xia et al., 2018; Pietri et al., 2018).

### 1.3 Symbiont-mediated pesticide detoxification in insects

Bacterial symbionts have been demonstrated to play key roles in a varied of host physiologies in insects including an active role in pesticide resistance (Blanton and Peterson, 2020). The first documented occurrence of symbiont-associated pesticide resistance was in the apple maggot, *Rhagoletis pomonella* (Boush and Matsumura, 1967). *Pseudomonas melophthora*, a bacterial symbiont of *R. pomonella,* is capable of degrading six organophosphate pesticides (Boush and Matsumura, 1967). After this observation, it took nearly fifty years for another documented case of symbiont-mediated pesticide detoxification to be reported. In 2012 a soil *Burkholderia* isolate was found to associate with both the bean bug, *Riptortus pedestris* along with the allied stink bug, *Cavelerius saccharivorus* and this association increased host tolerance fenitrothion in both lab and field assays (Kikuchi et al., 2012). Typically, symbiosis with detoxifying bacteria emerges in hosts following exposure to a pesticide as was seen in the brown plant hopper, *Nilaparvata lugens* and the gut symbiont, *Arsenophonus sp.* (Pang et al., 2018). This pattern has been supported in many examples across insect taxa.

### 1.4 Insect-associated microbes may demonstrate utility in bioremediation before control failure

The body of evidence that insect symbionts can detoxify and neutralize pesticides is substantial (Sharma et al., 2018; Blanton and Peterson, 2020). In many ways, this concept is intuitive. Insects are the targets of much of the agricultural and industrial pesticide use globally, so by exposing insects and their symbionts to pesticides we have selected for resistant, or at least tolerant, taxa (Berticat et al., 2002; Kikuchi et al., 2012; Almeida et al., 2017; Soltani et al., 2017; Pang et al., 2018; Pietri et al., 2018; Xia et al., 2018). However, most of our attention has been focused on screening pesticide-resistant insects to identify bioremediating bacteria (Almeida et al., 2017). This approach is certainly viable, and as mentioned above, is a reliable mechanism for isolating and identifying bacteria capable of pesticide degradation. In addition, we propose investigating the microbiota of pesticide susceptible insect targets for tolerant or resistant bacteria. The rationale for this is two-fold, 1) this may allow for the identification of resistance in a population where resistance is below the detection limit or does not rise to the level of control failure and 2) identifies bacteria with innate tolerance or abilities to transform pesticides that may not be in sufficient abundance to confer resistance to their hosts.

Due to their role as targets of pesticide application, their close association with symbiotic microbes, and their ubiquity in urban, suburban, and rural landscapes insects are a practical and promising source for microbes with bioremediation potential. Sourcing insect-associated bacteria for bioremediation potential offers additional advantages. These bacteria typically grow optimally in the same environments their hosts thrive in and, importantly, are in the same environment where pesticides are deployed and persist (Ahsan and Shimizu, 2021). Some symbiotic bacteria can facultatively associate with their insect host and live independently in the soil (Kikuchi et al., 2012; Polin et al., 2015; Heyworth et al., 2020; Zytynska et al., 2021). This means that these bacteria may be present in the same ecosystems where bioremediation is needed, but simply need to be nurtured to increase in abundance and speed up the remediation of their environment. Based on the insecticide-bioremediating bacteria identified to date, they are typically not plant or human pathogens which will be important if they are to be intentionally inoculated in the environment. These bacteria may also be useful in other applications, like protecting pollinators and non-target species from pesticides.

### 1.5 Termite symbionts as a fount of useful enzymes in biotechnology applications

Termites are perhaps best known for their association with symbiotic microbes. While initially recognized for their importance in wood digestion (Cleveland, 1925), termite symbionts are now known to contribute to nearly every physiological process in their hosts (Scharf and Peterson, 2021). Symbionts in the termite gut are associated with pathogen defense (Rosengaus et al., 2014; Peterson and Scharf, 2016), hormone modulation and regulation of caste (Sen et al., 2013; Sapkota et al., 2021), fitness (Rosengaus et al., 2011), and behavior (Duarte et al., 2018).

Some termite symbionts and symbiont-derived products are also amenable to laboratory rearing. Recombinant enzymes from microbes in this system are active at a wide range of temperatures and pH and are stable for days at room temperature (Coy et al., 2010; Zhou et al., 2010; Wang et al., 2012). We view these characteristics as assets for technology transfer. For these reasons, we propose that termites are an insect of interest when investigating insect-associated microbes for broad use in biotechnology.

Gut microbiota are critical to the biology of termites. Additionally, these symbionts have been useful source enzymes for applied purposes (Scharf, 2015). Imidacloprid has been used as a standard treatment for termite infestations since its release in the 1990 s (Hadi et al., 2020). Given these facts, we hypothesized that there are imidacloprid-tolerant bacteria in the gut of *R. flavipes* pesticide-naïve workers. To approach this, we started by identifying isolates from the termite gut predisposed to a tolerant phenotype and selected for this tolerant phenotype via a 10-day continuous culture assay. We then tested impact of the extended exposure regime on the tolerant phenotype via a 24-h growth assay comparing the parent strain to the daughter strain to a range of increased pesticide concentrations and assessed relevant the protein profiles of these same bacteria. Finally, we identified the unknown *R. flavipes* gut symbiont via whole genome sequencing. This study highlights that prolonged exposure to pesticides within the environment can be selected for symbionts capable of tolerating higher concentrations of pesticides.

## 2 Materials and methods

### 2.1 Termite collection and laboratory rearing

As described in previous studies (Peterson et al., 2015), *Reticulitermes flavipes* termites used in this project were collected on the Purdue University campus in West Lafayette, Indiana between May and July 2016. Colonies were reared in darkness at 23 ± 2°C with ∼40% relative humidity and provided with pine wood shims and brown paper towels as a food source. Relevant to this project, these termite colonies had no direct exposure to pesticides of any kind after collection.

### 2.2 Isolation of bacteria inherently imidacloprid tolerant bacteria from the termite gut

Following a similar proceed to Peterson et al. (2015), termite workers were dissected under a dissecting microscope using equipment sterilized with 70% ethanol. First, we dissected in a droplet of sterile sodium phosphate buffer (100 mM sodium phosphate, 5 mM calcium chloride at pH 7) and pooled 10 *R. flavipes* worker whole guts and suspended them in 100 μL of the same buffer. Then we used a micropestle to homogenize the guts in the same microcentrifuge tube containing sodium phosphate buffer before serially diluting with sterile water (10^−2^–10^−12^). Then those diluted gut contents were spread onto plates containing Brain-Heart Infusion agar (BHI) in triplicate and incubated at 27°C overnight. Bacteria grown from termite gut contents were aseptically sub-cultured from this mixed-culture plate onto fresh BHI agar plates subsequently until all isolates were in pure, monoculture. These isolates were screened for inherent imidacloprid tolerance.

Briefly, 5 mL Luria Broth (Miller) (LB) were inoculated with a single colony of a given isolate and incubated overnight at 23°C while shaking at 150 rpm. Optical density (OD, 600 nm) readings of overnight cultures were taken, cell densities were standardized and used to inoculate either 5 mL of peptone water (Thermo Fisher), a minimal growth medium or 5 mL of peptone water with the addition of the recommended concentration of formulated pesticide containing imidacloprid (BioAdvanced Tree and Shrub by Bayer, 2.562 μL/mL). Liquid cultures were then grown in an incubator at 23°C for 48 h (hr) with OD readings taken at 24 h and 48 h shaking at 150 rpm. These experiments were performed in triplicate for each isolate and calculated Chi-square values in R v 4.0.3 to evaluate the impact of imidacloprid on bacterial growth. A total of 33 isolates were screened.

### 2.3 Continuous culture assay

From this initial screening, we chose one isolate, Rf10 to subject to a prolonged exposure regime due to their initial imidacloprid tolerance. Similar to other studies interested in artificially enhancing bacterial phenotypes (Swenson et al., 2008; Waters et al., 2015), Rf10 was grown in triplicate continuously for 10 days in 1.5 mL of liquid peptone water containing the recommended rate of formulated pesticide containing imidacloprid. All isolates were sub-cultured every 12 hours and growth measurements (OD at 600 nm) were recorded concurrently. This resulted in a total of 20 passages of each isolate. Glycerol stocks were made on day 5 and day 10. Day 10 isolates (artificially selected isolates) and day 0 isolates (original isolates) were then struck out onto brain heart infusion agar plates for additional characterization. Based on our preliminary data, Rf10 has an approximate doubling time of 38 min, meaning this assay ran for >300 generations.

To compare growth in pesticide-rich media before and after continuous culture, overnight cultures of all parent and daughter isolates were standardized as previously described. A standardized, estimated cell count was added to each of three tubes containing 50 mL of peptone water and three tubes containing 50 mL peptone water containing the formatted insecticide (2.562 μL/mL). All tubes were incubated at 23°C and shaking at 150 rpm. Growth (OD 600 nm) measurements were taken every 2 hours for 12 h starting at the 4 h mark and then a final 24 h timepoint was taken. For statistical analysis, we use repeated measures ANOVA. Significance was assessed at a α = 0.05. All analysis and figures were generated using R 4.0.3.

### 2.4 Pesticide concentration gradient growth assay

All isolates, original and artificially selected, were primed by growing overnight cultures in a 1x pesticide concentration at a volume of 20 mL of peptone water. Bacterial cultures were standardized and sub-cultured into 20 mL liquid cultures at concentrations ranging from 1x (2.56 μL/mL), 2.5x (6.41 μL/mL), 5x (12.81 μL/mL), and 10x (25.62 μL/mL) of the formulated rate of imidacloprid-containing pesticide (BioAdvanced Tree and Shrub by Bayer). Cultures were incubated at 23°C and shaking at 150 rpm. Growth measurements (OD 600 nm) were recorded, and cell pellets collected at 24 and 48 h.

### 2.5 Soluble protein extraction, quantification, and visualization

Soluble protein was extracted from pelleted bacteria, via centrifugation at 6,000 rpm for 5 min, using the ThermoScientific B-PER reagent with a modified procedure. Briefly, the pelleted bacteria described above were resuspended in 500 μL B-PER reagent with 2 μL each of lysozyme and DNase A added and incubated for 15 min at room temperature. Soluble proteins were collected in the supernatant following centrifugation at 15,000 × G for 5 min.

Protein suspended in the supernatant was quantified using the Pierce Coomassie (Bradford) Protein Assay Kit to manufacturer’s specifications for a 96-well microplate format. After 10 min of color development, endpoint readings were taken at 595 nm using a BioTek Synergy H1 microplate reader. Sample protein estimations were calculated based on a standard curve of bovine serum albumin (ThermoScientific).

To visualize protein profiles, samples were standardized to 2 μg and mixed 5:1 with 6X Laemmeli sample buffer (ThermoScientific) and then boiled for 5 min. Samples were loaded into a 4%–20% Mini-Protean TGX Precast Protein Gel (BioRad) with 5 μL Spectra Multicolor Broad Range Protein Ladder (ThermoScientific) for reference. Gels were run at 100 V for 60 min, stained overnight with GelCode Blue Safe Protein Stain (ThermoScientific), destained in 10% acetic acid for 6 h, and imaged using the BioRad ChemiDoc MP imaging system. Densitometry analysis was performed using the gel analysis tools in ImageJ (https://imagej.nih.gov/ij/download.html).

### 2.6 Proteomics analysis and analysis

A band which was consistently associated with the parent isolate but decreased in abundance in artificially selected isolates grown in high pesticide concentration was cut from the gel using a sterile razor blade. The slice was placed in a sterile 1.5 mL microcentrifuge tube and sent to Creative Proteomics for analysis. Briefly, the gel slice was subjected to digestion with trypsin and were analyzed with nanoflow liquid chromatography tandem mass spectrometry (Nano LC-MS/MS). Nano LC-MS/MS analysis was carried out using a Thermo Fisher Ultimate 3,000 nano UHPLC system. The full scan was performed between 300–1,650 m/z at the resolution 60,000 at 200 m/z, the automatic gain control target for the full scan was set to 3e6. The MS/MS scan was operated in “Top 20” mode using the following settings: resolution 15,000 at 200 m/z; automatic gain control target 1e5; maximum injection time 19 m; normalized collision energy at 28%; isolation window of 1.4 Th; charge sate exclusion: unassigned, 1, >6; dynamic exclusion 30 s.

### 2.7 Genome sequencing, assembly, and annotation

To identify the isolate that had demonstrated inherent tolerance to imidacloprid and develop hypotheses about the mechanism of this tolerance, we submitted the Rf10 isolate for sequencing. Pelleted overnight cultures of bacteria were shipped on dry ice to CD Genomics (Shirley, NY) for sample processing and sequencing. Briefly, Genomic DNA was extracted with the SDS method. The harvested DNA was detected by the agarose gel electrophoresis and quantified by QubitR 2.0 Fluorometer (Thermo Scientific). A total amount of 200 ng DNA per sample was used as input material for the library preparations. Qualified genomic DNA was fragmented using Covaris g-TUBE devices and are subsequently repaired by treating the sample with a DNA-damage repair and A-tailing mix. Then adapters incorporating a unique index are ligated to each end. The libraries with a fragment size ∼470 bp are selected using BECKMAN AMPure XP Beads. Library quality was analyzed by Qubit and real-time PCR System, and average fragment size was estimated using an Agilent 2,100 Bioanalyzer. The whole genome was sequenced using Illumina Novaseq/Hiseq PE150 platform. Resulting paired-end reads were assembled using SPAdes (Prjibelski et al., 2020). The assembly was evaluated using QUAST and annotated using DFAST (Gurevich et al., 2013; Tanizawa et al., 2018). The assembly is available via NCBI at Accession Number SAMN22569637. Additionally, to compare the Rf10 genome to other bacteria, we queried freely available data through the Nation Center for Biotechnology Information (NCBI) and restricting the species output to either *Chryseobacterium* or *E. coli* K12 (Sayers et al., 2022).

## 3 Results

### 3.1 Some termite symbionts demonstrate inherent imidacloprid tolerance

To determine if isolates were inherently tolerant to imidacloprid, they were grown in media with and without the pesticide. Of the 33 isolates screened, most were unable to grow in the minimal media or never grew in the presence of imidacloprid (data not shown). After initial screening, we identified seven termite gut symbionts that could grow in the presence of imidcloprid. Growth at 24 h and 48 h post-inoculation was taken for three replicates of each of the seven isolates growing in minimal media with 1X imidacloprid and normalized to the growth of the same isolate in minimal media lacking imidacloprid (Table 1). While there were several isolates that grew better in minimal media with imidacloprid, Rf10 had a quantitatively higher OD_600 nm_ at both time points.

**TABLE 1:** Initial screening yielded several inherently imidacloprid-tolerant termite gut isolates. Average growth is reported as OD_600nm_ of a given isolate in minimal media with imidacloprid—OD_600nm_ of a given isolate in minimal media without imidaclorpid (OD^PWI^- OD^PW^ = ΔOD). Where ΔOD is negative, the isolate grew better in media lacking pesticide. Where ΔOD is positive, the isolate grew better in media with pesticide. An * indicates a signifcant X^2^ value where we refute the expection that all isolates to grow the same in both media types and the isolate grew better in the pesticide condition.

	24 Hours	48 Hours
Isolate	Ave Δ OD 24 h	Ave Δ OD 48 h
Rf5	0.1*	0.02
Rf10	0.02	0.04*
Rf25	−0.03	0.02*
Rf26	0.03*	0
Rf27	−0.06	0.04*
Rf31	−0.02	0.06*

### 3.2 Following constant culture in imidacloprid an inherently tolerant termite symbiont grows as well in media with and without the pesticide

**FIGURE 1:**
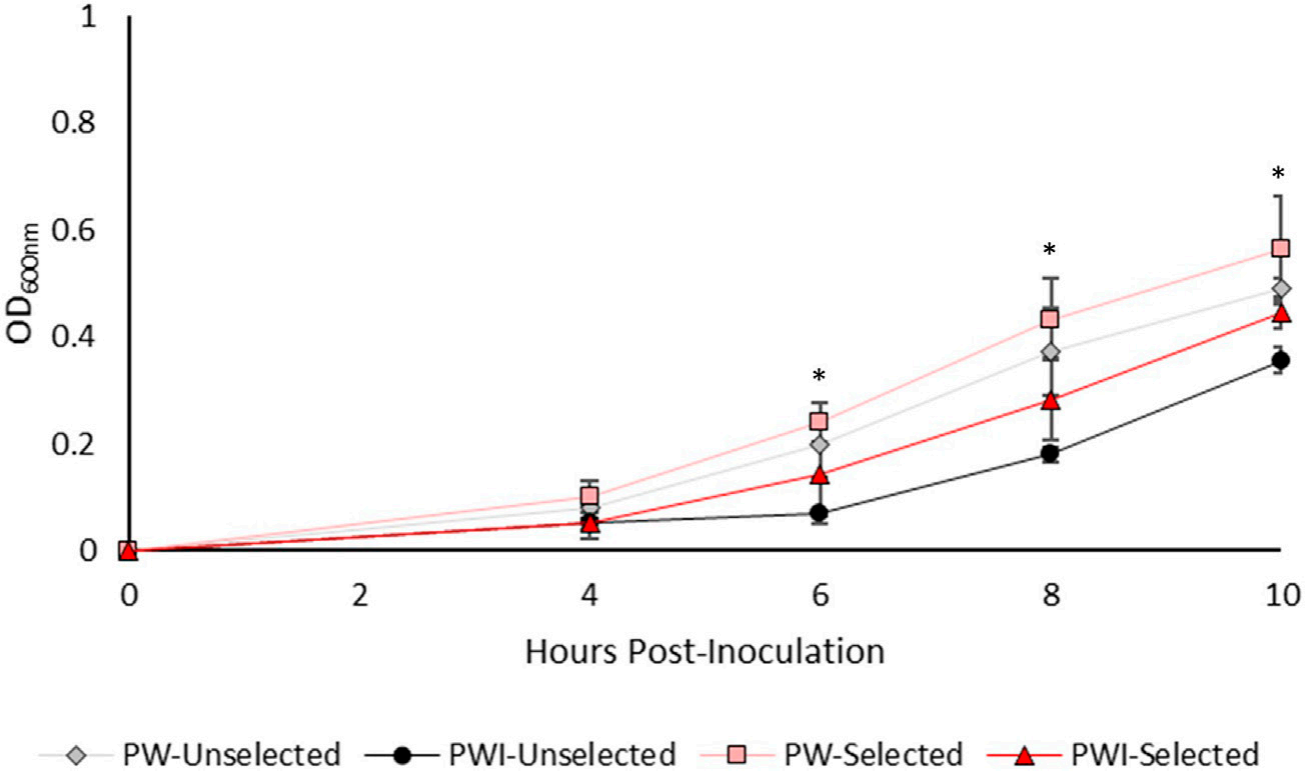
The imidacloprid-selected isolates of Rf10 exhibit similar growth patterns in imidacloprid to isolates grown in absence of imidacloprid. Three replicates of the parent isolate and the three artificially selected isolates were monitored for grow in minimal media with (PWI) and without imidacloprid (PW). We used repeated measures ANOVA to determine if selection regime or media type impacted Rf10 growth. An * indicates a timepoint where the unselected, parent isolate performed significantly worse in minimal media with pesticide compared to the isolates grown without pesticide (*p* < 0.05). The selected isolates were not statistically different from either the pesticide-free media.

After 10 days of constant culture in imidacloprid, we expected the selected, daughter isolates would perform better than their unselected parent isolates in the presence of imidacloprid. We assayed growth over 24 h, but cultures plateaued after ∼10 h. In the first 10 h of growth, the artificially selected isolates of Rf10 growing in imidacloprid are statistically indistinguishable from both the selected and unselected islates growing in pesticide-free media (Figure 1). In contrast, the parent isolate grew significantly less than Rf10 isolates in pesticide free media starting at 6-h post-inoculation (Figure 1). After 8 h, the unselected Rf10 isolate was growing significantly slower than all isolates (Figure 1). This demonstrates that after constant exposure to the pesticide, tolerance and/or growth rate increased in Rf10.

### 3.3 Constant exposure primes a termite symbiont to increased tolerance and differential enzyme regulation

**FIGURE 2:**
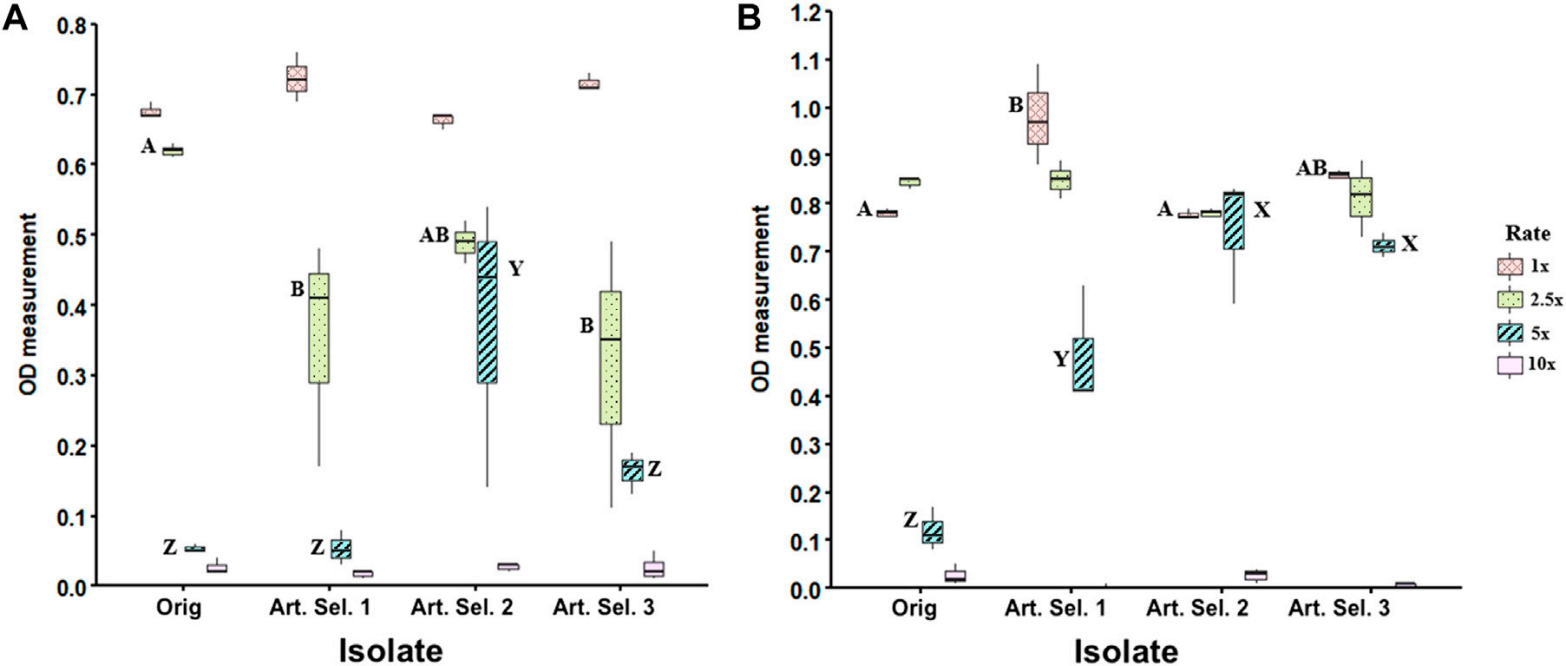
Extended exposure to 1X concentration of imidacloprid yields tolerance to higher concentrations relative to the parental isolate. Concentration gradient experiment in which the parent strain and three artificially selected strains (Art Sel-1, Art Sel-2, and Art Sel-3) were grown at four different concentrations (1x, 2.5x, 5x, and 10x) of imidacloprid in triplicate. Optical density measurements (600 nm) were taken at 24 h **(A)** and 48 h **(B)** timepoints. Boxes represent the differences in growth between the three replicates. Letters represent Tukey HSD *post hoc* analysis and were used to compare across isolates within a concentration, boxes with different letters are statistically different from each other. The 2.5 and 10X concentrations yielded no statistical differences across isolates, this is indicated by a lack of letters.

To determine the affect of a 10-day imidacloprid exposure on Rf10 tolerance of higher pesticide concentrations, we grew the parent and daughter isolates in a range of imidacloprid concentrations (1–10X) for 48 h. There is no significance between parent isolate and selected isolates in terms of mean growth at 24 h (Figure 2A). Results indicate that at 24 h isolate Rf10 can grow in up to a 5X (12.81 µL imidacloprid formulation/mL of medium) whether selected or unselected. Post hoc analysis further supports the data visualization at 24 h (Figure 2A) by confirming that selected isolates Rf10-1 and Rf10-3 are different from Rf10-0 and Rf10-2 at 2.5X the concentration (Figure 2A). However, Rf10-0 and Rf10-2 are not statistically different. Strikingly at 5X the concentration, Rf10-2 is significantly significant compared to the three other isolates.

After 48 h of incubation, there is a significant difference (*p* < 0.05) in mean growth at the 1x and 5x the formulation rate (Figure 2B). At 1x, the first selected isolate (Rf10-1) grew more than the unselected isolate (Rf10-0) and selected isolate two (Rf10-2). All selected isolates (Rf10-1, 10–2, and 10–3) exhibit significantly more growth than the unselected parent (Rf10-0) in the medium containing 5x formulation rate (*p* < 0.00001) (Figure 2B). All isolates performed similarly in peptone water containing 2.5x the rate of pesticide (Figure 2B). In the medium containing the highest concentration of pesticide (10x), no isolate, regardless of selection, had significant growth (Figure 2B).

**FIGURE 3:**
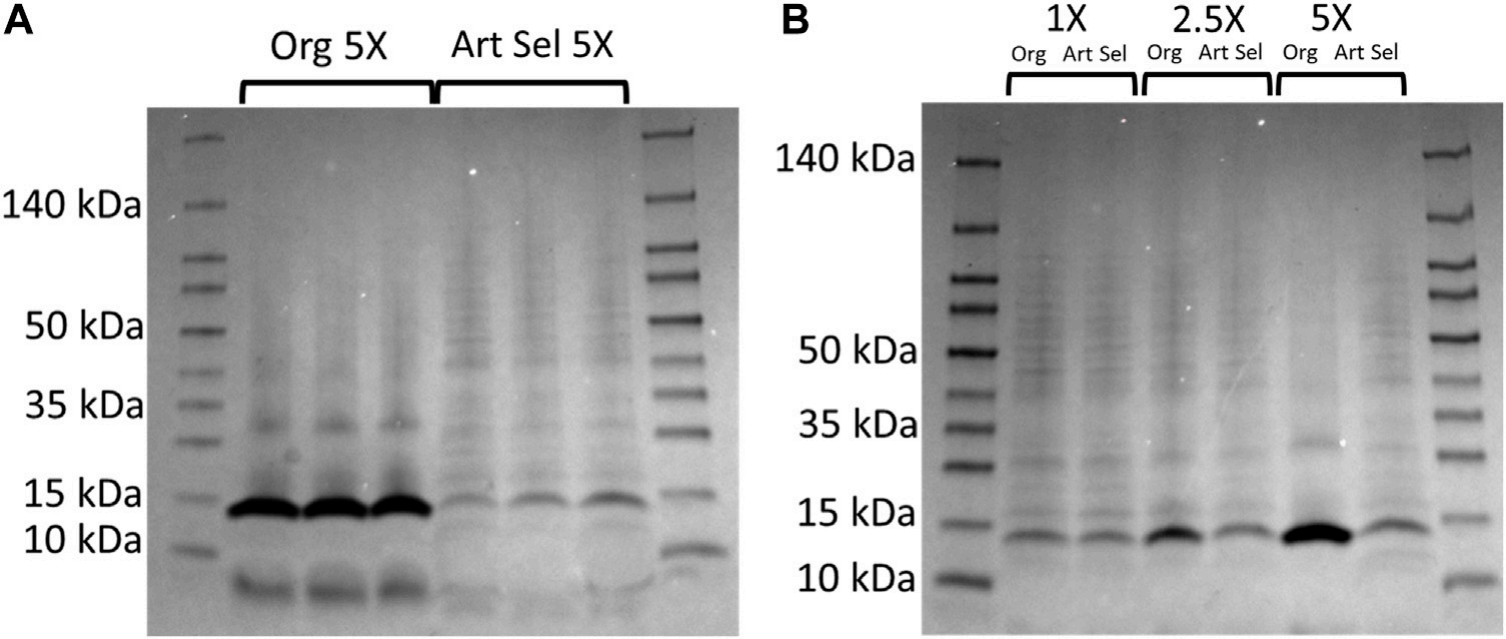
Original and artificially selected isolates and selected Rf10 bacteria exhibit distinct protein profiles in media containing high concentrations of formulated imidacloprid pesticide. **(A)**. Protein profiles of the original (Lanes 2–4) and a representative 10-day artificially selected isolate (Lanes 4–7). Rf10 isolates grown for 48 h in peptone water with 5X imidacloprid. Lanes 2-7 of this 4%–20% polyacrylamide gel contains 2 μg of protein and lanes 1 and 8 contain 5 μL molecular ruler for reference. **(B)**. Protein profiles of the original isolate (lanes 2, 4, and 6) and a representative 10-day artificially selected isolate (lanes 3, 5, and 7) Rf10 at 1X (lanes 2-3), 2.5X (lanes 4-5), and 5X (lanes 6-7) the formulation rate of imidacloprid in peptone water. Two micrograms of total protein were run for each bacterial sample (lanes 2–7) and lanes 1 and 8 contain 5 μL molecular ruler for reference.

Additionally, from these cultures protein profiles were assessed at the 48 h timepoint. At the 5X concentration were observed a 14 kDa band that is present and abundant in all replicates of the parent isolate, but is significantly reduced in the daughter isolates (Figure 3A). Using densitometry, we determined that this band 4.5x larger than the same band in the unselected bacteria (two-tailed, paired *t-*test *p* = 0.007). Additionally, a less pronounced band at <10 kDa is also more abundant in the parent compared to daughter isolates (two-tailed, paired *t-*test *p* = 0.019). Then we compared parent and daughter isolates’ protein profiles at the three imidacloprid concentrations where the bacteria were able to grow (1X, 2.5X, and 5X) and saw the same ∼14 kDa band increases in abundance in the parent isolate proportionally with the concentration of imidacloprid in the media (Figure 3B).

To identify this band, it was excised and sent for Nano LC-MS/MS analysis. This 14 kDa protein was identified as protein-L-isoaspartate *O*-methyltransferase. Protein-L-isoaspartate *O*-methyltransferase is an oxidoreductase. Based on protein profiles, this protein-L-isoaspartate *O*-methyltransferase is inversely associated with imidacloprid tolerance. Using the densitometry analysis, we correlate the intensity of this 14kDA band with the concentration of imidacloprid in the medium. In the parent isolate, this relationship was tightly correlated (*R* ^*2*^ = 0.99), but the same was not true for the daughter isolates (*R* ^*2*^ = 0.14). Therefore, growth in the presence of imidacloprid in primed, artificially selected isolates is associated with a downregulation of protein-L-isoaspartate *O*-methyltransferase relative to the naïve, parent isolate.

### 3.4 Genome seqencing reveals this inherantly tolerant termite symbiont to be *Chryseobacterium*

Given its interesting imidacloprid-related phenotypes, we performed whole genome shotgun sequencing on Rf10 to determine its identity and the genetic information underlying these traits. Based on sequencing, this bacterium is a *Chryseobacterium.* Its genome is just over 5 Mbp and has a GC content of 36%; the resulting assembly consists of eight large contigs (>50,000 bp) and several smaller contigs (Table 2). In comparison to the two annotated *Chryseobactium* genomes annotated and available through NCBI, the Rf10 isolate of *Chryseobacterium* is similar in size, gene number prediction, and GC content (Table 2). In addition, this identification as a *Chryseobacterium* species is consistent with other traits of this isolate including a yellow/orange pigmentation, aerobic growth, and Gram-negative rod morphology.

**TABLE 2:** Comparative Genome Assembly Statistics. Assembly and genetic element comparison of the *Chryseobacterium* isolate in this study to two publicly available *Chryseobacterium* genomes available.

Assembly Statistic	Rf10 assembly	*Chryseobacterium indologenes* NCTC10796	*Chryseobacerium arthrosphaerae* LMY
Total Length (Mb)	5.1	4.9	5.4
# Contigs	12	1	1
# Contigs (≥ 5,000 bp)	9	1	1
# Contigs (≥ 50,000 bp)	8	1	1
Largest Contig (Mb)	2.2	4.9	5.4
GC (%)	36.06	37.2	37.9
N50	982,807	4,900,000	5,400,000
N75	751,693	--	--
L50	2	1	1
L75	3	--	--
#N’s per 100 bp	10.08	--	--
Gaps	3	--	--
# CDS	4,462	4,361	4,765
Genes assigned to COGs	1,162	--	--
rRNAs (5 S, 16 S, and 23 S)	3	18	18
tRNAs	77	90	92
tmRNA	1	3	3
Total Features	4,545	4,497	4,915

We also annotated the genome to identify genes encoding potential detoxification enzymes (Table 3) and pesticide bioremediation-related genes (Table 4). This *Chryseobacterium* isolate from the *R. flavipes* gut has a large number of α/β hydrolases compared to *E. coli* K12, but less than that of other *Chryseobacterium* species (Table 4).

**TABLE 3:** Summary of detoxification enzyme annotated in the *Chryseobacterium* sp. from *R. flavipes* worker gut. Enzyme class is noted on the right with the total count of genes detected in the Rf10 *Chryseobacterium* sp. genome. Numbers denoted in parentheses are a subset of their parent category. Here Rf10 is compared to known *Chyseobacterium* genomes, specifically *C. indologenes* and *C. arthrosphaerae* with annotated genomes available through NCBI. For comparison, the total gene count in each category for *E. coli* K12 is provided for reference. NA means that no genes with this specific annotation are listed.

*Enzyme*	*Rf10*	*Chryseobacterium*	*E. coli* K12
Catalase	2	3	2
Peroxidase	7	11-12	4
Redoxin	15	23	11
Ferredoxin	(3)	(1)	(8)
Peroxiredoxin	(4)	(3)	(1)
Thioredoxin	(8)	(19)	(2)
Phosphoesterase	4	1–5	0
Oxidase	3	12–15	10
Ferritin	2	2	2
Glutathione Peroxidase	2	1	1
Superoxide Dismutase	2	4	4
Thioesterase	3	11-12	3
Catalase-Peroxidase	1	1	NA

**TABLE 4:** Summary of pesticide detoxification-associated enzymes annotated in the *Chryseobacterium* sp. from *R. flavipes* worker gut. Enzyme class is noted on the right with the total count of genes detected in the Rf10 *Chryseobacterium* sp. genome. Numbers denoted in parentheses are a subset of their parent category. Here Rf10 is compared to known *Chyseobacterium* genomes, specifically *C. indologenes* and *C. arthrosphaerae* with annotated genomes available through NCBI. For comparison, the total gene count in each category for *E. coli* K12 is provided for reference. NA means that no genes with this specific annotation are listed.

Enzyme	*Rf10*	*Chryseobacterium*	*E. coli* K12
Esterase	3	3–6	1
Hydrolase (α/β)	27 (16)	157–176 (28–32)	17
Glucosidase	4	3-4	3
Phosphatase	2	34–43	47
Transferase	1	29–49	1
Dehalogenase	3	6–11	NA

## 4 Discussion

In this study, we found evidence supporting our hypothesis that imidacloprid-tolerant gut-symbionts in an insecticide naïve termite host. We identified one symbiont of interest as a belonging to the genus *Chryseobacterium.* This isolate of *Chryseobacterium* was found to not only have inherent tolerance to imidacloprid, but also this tolerance improved after 10-days of constant exposure. This improved tolerance was correlated with a decrease in the expression of protein-L-isoaspartate *O*-methyltransferase in artificially selected isolates relative to the parent isolate. Further, we annotated an increased number of α/β hydrolases in this isolate; these enzymes may be candidates for further inquiry as potential metabolic mechanisms for the observed lower stress response within *Chryseobacteria* spp. after prolonged exposure. Taken together this study highlights an important step in identifying bacteria with the potential to confer tolerance to their hosts before resistance emerges. Taking these nuanced relationships between insects and their gut microbiota into account where bacteria have the potential to be directly exposed to pesticides provides new sources of pesticide tolerant and bioremediating bacteria that may have previously gone undetected. This effect compounds when taking eusocial behaviors, such as coprophagia, within termites into account which provides the potential for gut microbes to be passed from parent to offspring over several generations.

### 4.1 Known associations between *Chryseobacterium* spp. and insects

*Chryseobacterium* isolates have been found in several arthropod taxa (Dugas et al., 2001; Walenciak et al., 2002; Campbell et al., 2004; Burešová et al., 2006; Kämpfer et al., 2010; Lee et al., 2016; Blow et al., 2017; Reetha and Mohan, 2018; Skowronek et al., 2020). In ticks, the association with *Chryseobacterium* is an antagonistic one, where the bacterium can penetrate the gut and induce mortality in ∼72 h (Burešová et al., 2006). However, in white-spotter flower chafer larvae, *Chryseobacterium* sp. colonize their guts and are less likely to trigger an immune response (Lee et al., 2016). *Chryseobacterium* spp. are also members of the normal gut flora in the American cockroach, the watermilfoil moth, the pink stem borer, two mosquito species, the olive fruit fly, and the common cockchafer (Dugas et al., 2001; Walenciak et al., 2002; Campbell et al., 2004; Burešová et al., 2006; Kämpfer et al., 2010; Lee et al., 2016; Blow et al., 2017; Reetha and Mohan, 2018; Skowronek et al., 2020).

In *Periplaneta americana*, the abundance of a cultivable *Chryseobacterium* isolate was correlated with a high-fiber diet (Dugas et al., 2001). When another species of cockroach, *Blattella germanica,* was treated with antibiotics they suffer increased susceptibility to pesticides indicating a link between the cockroach microbiome and host pesticide tolerance (Pietri et al., 2018). Cockroaches are an important model system used to understand pesticide resistance mechanisms and, considering their evolutionary relationship, the association of *Chryseobacterium* spp. with *R. flavipes* is to be expected (Inward et al., 2007). In fact, a *Chryseobacterium* isolate from another member of Rhinotermitidae, *R. aculabialis*, was recently described as well (Zhao et al., 2017). However, the present study would be the first to directly link a *Blattodea*-associated *Chryseobacterium* to an enzyme-based stress response which decreases with exposure over time. Taken together, it will be important to measure whether this change in stress response could indicate a shift in bacterial metabolism resulting in host tolerance to Imidacloprid by *Chryseobacterium* spp. *in vivo*.

### 4.2 *Chryseobacterium* metabolism links pesticides and stress response

*Chryseobacterium* was initially a classified within the Flavobacteriaceae and recently has been reclassified as a distinct group (Son et al., 2022). *Flavobacterium* spp. and subsequently *Chryseobacterium* spp. have been known for both their free-living capacity and their potential as opportunistic pathogens (Cimmino and Rolain, 2016; Saticioglu et al., 2021; Zhang et al., 2022). Several strains of both groups have been documented as carriers of antibiotic resistant genes and still others have been documented as having chemical bioremediation potential (Sethunathan and Yoshida, 1973; Chaudhry and Huang, 1988; Nayarisseri et al., 2015; Cimmino and Rolain, 2016; Park et al., 2022). In the context of the genus *Chryseobacterium*, the Rf10 isolate is rather typical. With similar size genome, number of predicted genes, and GC content to other medically important species, like *C. indologenes* and *C. arthrosphaerae,* deposited in the NCBI genome archive (PRJEB6403 and PRJNA719258 respectively). A recent comparative study characterized genomes of seven *Chryseobacterium* spp. from environmental and animal-associated samples demonstrates more diversity in the genomes of congeners (Matu et al., 2019). Genomes of the *Cryseobacterium* isolates in the comparative study range from 3.7–5.2 Mb and 33.6%–37.1% GC, but no gene annotation or prediction effort was performed (Matu et al., 2019). The opportunistic pathogens’ genomes described above show similar numbers of total genes but have markedly more copies of important detoxification enzymes like peroxidase, thioredoxin, thioesterase, oxidase, hydrolase, phosphatase, transferase, and dehalogenase (Table 2; Table 3; Table 4). This could be due to the overall quality of the genome assembly for the Rf10 *Chryseobacterium* isolate. In contrast to the complete genome sequences in these other species, our assembly still has gaps and has not been assembled into a single contig. Additional time and funding could allow for long-read sequencing efforts and improvement of the genome assembly overall. However, it could also be that the differences in niches, human pathogens *versus* termite gut symbionts, could account for differences in enzyme copy numbers.

In an analysis of empirical studies, we found that some *Chryseobacterium* strains are known to degrade carbofuran using monoxygenases and hydrolases and glyphosate using enzymes in the shikimate pathway (Taguchi et al., 1989; Park et al., 2022; Zhang et al., 2022). Protein-L-isoaspartate *O*-methyltransferase (PMIT) has been documented as a common stress response (Chavous et al., 2001; Kindrachukt et al., 2003). In *Drosophila*, this protein was associated with extended life due to its function in oxidative damage repair in cells (Chavous et al., 2001). In *Escherichia coli*, overexpression of PMIT was positively correlated with higher heat tolerance (Kindrachukt et al., 2003). Importantly, the mechanism of stress tolerance in *E. coli* was independent of PMIT’s methyltransferase activity (Kindrachukt et al., 2003). In our results, we see a decrease of PMIT levels over time as our isolate has more time to adjust to pesticide-containing media and this could be due to a latent secondary metabolism response. Changes to the metabolome following prolonged exposure has been documented in species of both *Flavobacterium* and *Chryseobacterium* (Taguchi et al., 1989; Park et al., 2022). Therefore, it is possible to say that a delayed secondary metabolism response could explain the lower PMIT levels seen in the artificially selected isolates, though exploration of response is required.

### 4.3 The termite gut as a hot bed of biotechnology potential

Even before this discovery of imidacloprid-tolerant gut bacteria, termites have been a fountain of potential for both pest control and industrial applications via gut enzymatic activity (Scharf, 2015). The interdependence between termites and their resident microbiota has led to the exploitation of many bacteria for practical applications. The most conspicuous of these is the investigation of termite-associated microbes for their utility in bioethanol production. Both bacteria and protists isolated from the termite gut have been scrutinized for their production of highly efficient cellulases, hemicellulases, and ligninases (Scharf, 2015). Importantly, glycosyl hydrolases, endoglucanases superoxide dismutases, glutathione peroxidases, ligninases, aldo-keto reductases, and xylose isomerase have been patented for use in industrial biofuel production pipelines (Lam and Mathur, 1999; Scharf et al., 2013; Katahira et al., 2014; Scharf and Sethi, 2016; Scharf and Sethi, 2016; Brevnova et al., 2019).

Enzymes derived from or targeted against the termite gut symbionts have also been proposed for practical uses more than just their wood-digesting abilities, including pest control and antifungal solutions (Bachelet et al., 2015; Scharf and Peterson, 2018; Scalzi et al., 2019; Mueller et al., 2020). With more characterization, it is possible that enzymes from from this *Chryseobacterium* sp. isolate could be exploited for *ex vivo* bioremediation efforts. If the downregulation of the protein-L-isoaspartate *O*-methyltransferase is important for hydrolysis of imidacloprid by another enzyme pathway, perhaps producing mutant lines of termite *Chryseobacterium* sp. that are deficit in that enzyme may further improve pesticide tolerance or turnover. By using insects as dispersal vectors for a dual microbial pesticide and bioremediating organism, organizations could cut down on labor and time as well as approach two individual agricultural problems simultaneously.

### 4.4 Potential for application of termite associated *Chryseobacterium* spp. in pesticide bioremediation

In terms of bioremediation, several strains of *Chryseobacterium* spp. isolated from agricultural soil have been found to degrade pesticides from different families and have been proposed as potential candidates for bioaugmentation projects (Ning et al., 2010; Qu et al., 2015; Silambarasan and Abraham, 2020). There also is evidence to suggest that *Chryseobacterium* can adapt to the presence of new pesticides quickly (Zhao et al., 2016). This is supported by the data in our study which indicates a protein level change within just 10 days of exposure and resulting in a tolerance five times higher In artificially selected strains compared to the original strain. Due to their aerobic nature, ease of cultivation, suite of detoxification enzymes, and repeated association with pesticide tolerance and turnover, we propose that *Chryseobacterium* spp. should be invested in for their bioremediation potential. While more data is needed, these bacteria isolated from agricultural soils and ecologically relevant insect hosts have abundant potential.

Remediating microorganisms that have been specifically modified to bioremediate a particular pesticide presents a unique potential for marketable microbial treatments for widely used agricultural pesticides. Much like oil-degrading bacteria have been used to bioremediate oil spills, if timed correctly, pesticide degrading bacterial applications could aid in limiting environmental accumulation of pesticides and reduce pesticide runoff into the surrounding environments while also reaping the benefits of pest control towards target insects (Gentili et al., 2006). Thus, lowering the half-lives of pesticides within the environment thereby aiding in the mitigation of safety hazards poised to field workers by pesticides. Based on our research, *Chryseobacterium* and others like it represent a starting point for these future developments in the field of agricultural biotechnology.

## 5 Conclusion

In this study, we have characterized a pesticide-tolerant symbiont within a susceptible insect host. While insect-associated bacterial tolerance and/or detoxification of insecticides has become well-established, no study has identified such bacteria in the termite gut. We have further identified the symbiont to be a member of the *Chryseobacterium* genus. Our study highlights the ability of this symbiont to adapt to tolerate up to 5x the pesticide concentration over a short time period of exposure highlighting its relevance to the field of biotechnology as a possible bioremediating organism. The potential exploitation of termite-associate symbionts has vast potential to improve the long-term efficacy of pesticides in both agriculture and urban pest management via biotechnology facilitated stewardship. Termites are a well-studied host organism, and their microbiome and physiology has been utilized before in studies related to second generation biofuel production. Additionally, knowing that it is possible to have a susceptible host harbor a tolerant symbiont could be one of the keys to anticipating and monitoring the progression of insecticide resistance in host populations as well as the development of new bioremediating techniques.

## Data Availability

The datasets presented in this study can be found in online repositories. The names of the repository/repositories and accession number(s) can be found below: https://www.ncbi.nlm.nih.gov/, SAMN22569637.
